# A Novel and Integrated Digitally Supported System of Care for Depression and Anxiety: Findings From an Open Trial

**DOI:** 10.2196/46200

**Published:** 2023-07-24

**Authors:** Kate Wolitzky-Taylor, Richard LeBeau, Inna Arnaudova, Nora Barnes-Horowitz, Elizabeth Gong-Guy, Scott Fears, Eliza Congdon, Nelson Freimer, Michelle Craske

**Affiliations:** 1 Department of Psychiatry and Biobehavioral Sciences University of California - Los Angeles Los Angeles, CA United States; 2 Department of Psychology University of California - Los Angeles Los Angeles, CA United States; 3 Greater Los Angeles Veterans Administration Los Angeles, CA United States

**Keywords:** depression, anxiety, cognitive behavioral therapy, digital mental health, stratified models of care, model of care, care model, depressive, mental health, CBT, psychotherapy, university, postsecondary, student, college, service delivery, care system, system of care, mHealth, eHealth, online support, student mental health

## Abstract

**Background:**

The global burden of anxiety and depression has created an urgent need for scalable approaches to increase access to evidence-based mental health care. The Screening and Treatment for Anxiety and Depression (STAND) system of care was developed to meet this need through the use of internet-connected devices for assessment and provision of treatment. STAND triages to level of care (monitoring only, digital therapy with coaches, digital therapy assisted by clinicians in training, and clinical care) and then continuously monitors symptoms to adapt level of care. Triaging and adaptation are based on symptom severity and suicide risk scores obtained from computerized adaptive testing administered remotely.

**Objective:**

This article discusses how the STAND system of care improves upon current clinical paradigms, and presents preliminary data on feasibility, acceptability, and effectiveness of STAND in a sample of US-based university students.

**Methods:**

US-based university students were recruited and enrolled in an open trial of the STAND system of care. Participants were triaged based on initial symptom severity derived from a computerized adaptive test and monitored over 40 weeks on anxiety, depression, and suicide risk to inform treatment adaptation and evaluate preliminary effectiveness.

**Results:**

Nearly 5000 students were screened and 516 received care. Depression and anxiety severity scores improved across all tiers (*P*<.001 in all cases). Suicide risk severity improved in the highest tier (ie, clinical care; *P*<.001). Acceptability and feasibility were demonstrated.

**Conclusions:**

STAND is a feasible and acceptable model of care that can reach large numbers of individuals. STAND showed preliminary effectiveness on all primary outcome measures. Current directions to improve STAND are described.

## Introduction

### Background

The public health burden of depression and anxiety is enormous and rising, with effects extending to unemployment, risky substance use, and of greatest concern, suicide. In particular, young adults have seen a substantial increase in anxiety, depression, and suicidal thoughts or behaviors over the past decade [1,2]. Mental health care systems, globally, face significant challenges in meeting the staggering need for care created by this rising public health burden. In particular, access to traditional forms of mental health care is limited by cost, time, effort, and stigma [3], and the number of clinicians trained in evidence-based treatments is grossly inadequate to meet current needs. The urgency of these needs has stimulated the development of 2 broad strategies for increasing access to care: stepped care approaches and digital provision of therapies.

### Increasing Access to Care Using Stepped Care Approaches and Digital Provision of Therapies

In stepped care models, typically, all patients begin with low-intensity treatments and only nonresponders are moved to the next (more intensive) step of care [4]. Low-intensity treatments often include digital or online therapies, with or without support, as multiple trials targeting anxiety and depressive disorders have shown that these treatments can be as effective as face-to-face therapy [5-8]. While this “fail-first” approach more efficiently allocates resources than traditional approaches that strive to provide the highest level of clinician-delivered care to all patients, it results in delays to more intensive treatment for those who need it [9], and such delays may lead to symptom worsening and increased disability [10], discouragement for new treatments after initial options fail [11], or dropout before a second course of treatment [12]. An emerging body of literature indicates that *stratified* models, in which individuals are matched to level of care based on predetermined criteria (typically current symptom severity), produce superior outcomes and are more cost-effective in treating depression and anxiety compared with fail-first stepped care models [13-16].

### Digital Technologies for Delivering Measurement-Based Care and Treatment Adaptation

The use of data collected from patients during the course of treatment for clinical decision-making (measurement-based care) has the potential to greatly improve outcomes but remains the exception rather than the rule [17]. Specifically, measurement-based care typically outperforms usual care on both symptom reduction and reduced dropout rate [18] and is associated with decreased costs and lower odds of patient deterioration during treatment [19]. Without measurement-based care, clinicians have difficulty identifying patients who are at risk for nonresponse or deterioration [20,21]. Consequently, interventions become reactive to “crisis” needs rather than being proactive. Further, decisions about when treatment has been successfully completed tend to be determined by when the patient or their provider “feels ready” to terminate therapy, which may lead to extending treatment far beyond the point when symptom gains have been attained and maintained.

Even when measurement-based care is utilized, guidelines on how to adjust treatment over time are lacking. As discussed, stepped care approaches typically personalize level of care *after* a patient has shown nonresponse. Waiting several months to make adaptation decisions could have dire consequences for individuals with depression and anxiety [22]. Moreover, typical stepped care approaches step up only for patients who remained actively engaged in treatment, thereby not addressing the critical issue of how to manage and engage individuals who prematurely discontinue treatment [23].

Furthermore, typical care models fail to fully consider changes in clinical status after the end of acute treatment; relapse is not uncommon (especially for depression), yet rarely are patients monitored in order to identify for whom reinitiation of care is warranted. By identifying those at risk for nonresponse or worsening of symptoms during or after treatment, adaptations can be made to increase treatment intensity or reenter treatment and prevent further worsening of symptoms and their potential consequences. Routine monitoring of clinical status enables dynamic adaptation of treatment as needed, which has the potential to improve effectiveness and reduce attrition, as patients may be more engaged in treatment when they are receiving what they need most at the time they most need it [24-26]. Adaptive interventions can also enhance cost-effectiveness by increasing the efficiency of service delivery and reducing downstream service costs [27].

### Digital Technologies for Identification and Management of Suicide Risk

Suicidal thoughts and behaviors are a major public health problem, particularly among young adults [28]. Rates of suicide attempts, nonsuicidal self-injury, and suicidal ideation increase markedly during adolescence [29-31], with suicide death rates increasing as youth move into adulthood. Across all adult age groups, the prevalence of serious suicidal thoughts is highest among young adults aged 18-25 (11%) [32]. There is a critical need to embed methods for detection of risk and strategies for suicide/self-harm prevention within systems of care, which go beyond relying on patients to reach out to providers or to call crisis lines, and beyond monitoring at frequencies that fail to capture tipping points toward high risk. Digital tools may be particularly useful in assessing and monitoring such risk, given that they can promote increased self-disclosure of sensitive topics compared with face-to-face assessment and allow for more rapid, in-the-moment identification and response [33].

### STAND: A Novel, Scalable, Dynamic, Digitally Assisted Evidence-Based Solution to Mental Health Care Delivery

**Figure 1:**
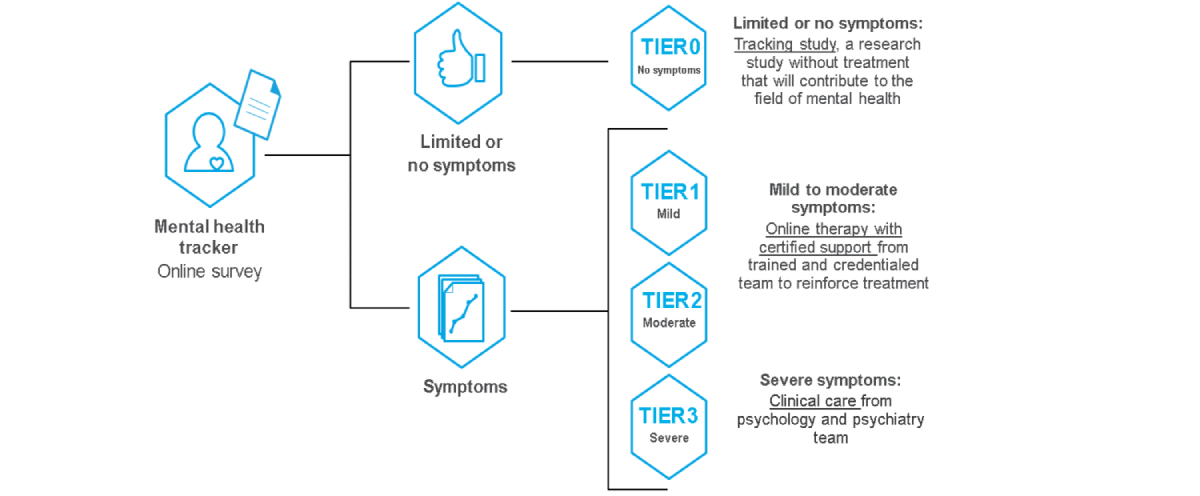
The STAND system of care. STAND: Screening and Treatment for Anxiety and Depression.

Screening and Treatment for Anxiety and Depression (STAND) is a stratified stepped care model that incorporates online screening, continuous symptom monitoring over ~10 months, and tiered treatment for anxiety and depressive symptoms, with ongoing suicide risk detection and management (Figure 1). After individuals complete a brief online adaptive assessment of symptom severity and suicide risk (selected modules from the Computer Adaptive Test for Mental Health [CAT-MH]; [34]), they are provided feedback, routed to an appropriate level of care, and scheduled for an orientation or intake session, all within minutes of completion of initial screening. The CAT-MH assessment continuously tracks symptoms (both during and after active treatment) to adapt treatment (ie, move to a higher level of care when symptoms worsened or to a lower level of care for maintenance and relapse prevention) and to rapidly detect and respond to signs of elevated suicide risk. For the open trial described herein, there were 4 levels of care, or tiers.

Tier 0 was for those with no or minimal symptoms of depression or anxiety on the initial CAT-MH assessment. These participants were offered the option to continue completing the CAT-MH biweekly [34]. Tier 1 was offered to those with mild depression or mild anxiety on the CAT-MH, and included digital therapy. Coaching was offered to tier 1 participants given evidence for its positive effects on retention and clinical outcomes from digital therapy [8]. Tier 2 involved digital cognitive behavioral therapy (CBT) with advanced (doctoral student) coaches, and was offered to individuals with moderate depressive symptoms or moderate to severe anxiety symptoms. Evidence-based psychological treatment with option for medication management was offered to those with severe depressive (or manic) symptoms or suicidality (tier 3). Core active ingredients of CBT (see the “Methods” section) were selected to match each participant’s problems areas and were delivered by clinical psychology graduate students. Therapy was complemented by protocolized medication management as needed (modified from the STAR*D trial; [35]), delivered by psychiatry residents.

**Figure 2:**
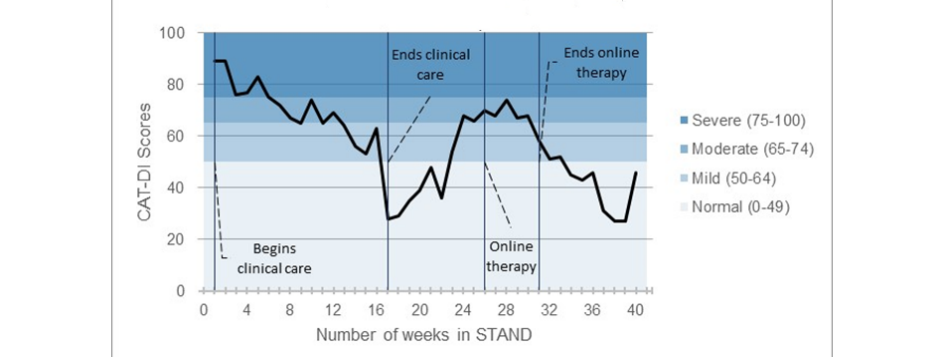
Treatment adjustment of a simulated patient in clinical care. CAT-DI: Computerized Adaptive Test-Depression Inventory; STAND: Screening and Treatment for Anxiety and Depression.

Across all tiers, treatment was adapted according to weekly or biweekly CAT-MH scores (Figure 2). If participants’ scores indicated a need for a higher tier of care at any time, they were contacted to initiate switching to the appropriate level of care. Similarly, participants in tier 3 could be switched to a lower tier and were thus offered access to digital therapy materials when they completed a course of tier 3 treatment and symptoms had shown improvement. Moreover, within tier 3, clinicians used weekly CAT-MH scores to guide decisions regarding treatment strategy (such as switching from behavioral activation to cognitive restructuring).

Frequent monitoring of suicide risk was conducted using a standardized protocol for responding to risk in real time (see the “Measures” section for details). This approach to risk management was implemented for all levels of care within STAND. In addition to outreach, a positive suicidal triggering alert indicated adaptation to tier 3 if the participant was in a lower tier.

### Summary of STAND Improvements to Clinical Practice Paradigms

STAND improves standard clinical practice paradigms in several ways. First, STAND provides increased access to evidence-based care by utilizing digital tools for a large proportion of the patient experience from screening through service enrollment to service delivery and risk management. Second, the emphasis on adapting both the level of care and intervention type as needs change is a major improvement over existing approaches (eg, stepped care), which typically wait until nonresponse is established at predefined points that are months apart before offering an alternative, if at all [16]. Third, STAND focuses on maintaining gains and minimizing relapse, which are rare in systems of care, despite the many benefits to individuals and a health system [36]. Finally, the STAND program focuses on personalization of services based on continuous symptom monitoring and clinical decision-making (both with regard to tier triaging and adaptation as well as choice of treatment modality within tier 3, the highest level of care) that is expected to reduce attrition and improve symptomatic and functional outcomes [23].

This study aimed to develop and evaluate the preliminary acceptability, feasibility, and effectiveness of the STAND program in a large sample of college students in an open trial. It was hypothesized that participants would report high satisfaction with the program, and would show significant improvement in anxiety, depression, and suicide risk severity scores over the course of participation in STAND.

## Methods

### Participant Recruitment and Initial Triaging

Recruitment and enrollment into the open trial occurred between Fall 2017 and Spring 2020. All University of California, Los Angeles (UCLA) students were made aware of the screener through announcements from UCLA’s Chancellor, tabling at campus events, campus residence hall flyers, classroom announcements, web-based advertisements, banners, and email blasts. Students completed the CAT-MH online and received immediate feedback regarding their scores and recommended tier of care. Those who were interested in and eligible for the study and who provided informed consent were then scheduled for a baseline visit. At the baseline visit, research staff gathered pertinent information about treatment history and other demographics, after which participants were notified about how to start care in their assigned tier, with ongoing completion of the CAT-MH and other assessments over the course of 40 weeks.

### Inclusion/Exclusion Criteria

All matriculating UCLA students who were at least 18 years of age and had access to the internet were eligible to complete the initial screener. Additional inclusion criteria for screening were English fluency, willingness to install an app on their phone, and no plans (eg, upcoming long vacations or studying abroad) that would interfere with participation. Additional exclusion criteria following screening for those entering clinical care (ie, tier 3) were severe psychopathology requiring intensive day-treatment or inpatient care (eg, severe eating disorder or substance use disorder, multiple [2+] suicide attempts leading to hospitalization within the past 6 months, significant psychotic symptoms not part of a severe major depressive episode, or bipolar manic episode) and were determined at the initial assessment through further screening. Participants in tier 3 were also excluded if they were unwilling to provide a blood sample (for secondary analyses outside of the scope of this manuscript) or had a current psychiatrist or provider and were unwilling to transfer their care to the study team for the period that they will receive treatment in STAND.

### Interventions

#### Tier 0

Participants whose Computerized Adaptive Test-Depression Inventory (CAT-DI) and Computerized Adaptive Test-Anxiety Inventory (CAT-ANX) scores were in the normal range were offered tier 0 or monitoring of their status using biweekly CAT-MH.

#### Tier 1

Those whose depression or anxiety scores were in the mild range were offered tier 1 that included an evidence-based digital CBT called *ThisWayUp* [37]. *ThisWayUp* provides 6 lessons on CBT strategies for anxiety and depression including behavioral activation, cognitive restructuring, and exposure. This digital therapy program was selected, as randomized controlled trials have demonstrated evidence to support its efficacy [37,38]. Digital CBT was accompanied by the offer of up to 8 phone or video coaching sessions by extensively trained undergraduate student coaches (see the “Methods” section for details) to support the digital therapy material and to assist with troubleshooting (rather than to teach clinical content). As human support has been shown to increase efficacy and retention in digital therapies [8], coaching sessions were offered each week after participants independently completed a *ThisWayUp* lesson. Coach training had 4 sequential components (see [39] for details). Didactic, interactive classroom meetings and small-group coaching support meetings focused on content and general coaching skills. Discussions were held weekly with opportunities to practice coaching skills under close supervision and with small-group feedback, and to achieve core competencies in professionalism, diversity awareness, ethical standards, and reflective practice and self-care. Advancement to the Certified Coach status was required before coaches could provide video-chat coaching to study participants, under supervision from program faculty. Coaching was initially provided to participants in a group format, and later was provided in an individual 1-on-1 format.

#### Tier 2

Tier 2 was offered to those who scored in the moderate range for depression or moderate to severe range for anxiety. Participants were assigned to complete *ThisWayUp* and were strongly encouraged to attend weekly face-to-face, video-chat, or phone coaching sessions following their independent completion of each *ThisWayUp* lesson. In addition to being trained in *ThisWayUp,* advanced (doctoral students) coaches participated in the tier 3 clinical training (described below) to gain a deeper knowledge of cognitive and behavioral principles. Given their more advanced training, tier 2 advanced coaches had more flexibility to support the CBT material in the coaching sessions. They received weekly supervision by licensed clinical psychologists.

#### Tier 3

Evidence-based psychological treatment with option for medication management was offered to those with severe depressive (or manic) symptoms or suicidality. Evidence-based psychological treatments often consist of multicomponent packages of different cognitive and behavioral strategies, such as cognitive restructuring, self-monitoring, problem solving, relaxation training, assertiveness training, or exposure therapy. Such combination packages prohibit understanding of which ingredient was most accountable for therapeutic change for each individual and the processes responsible for therapeutic change [40,41]. Approaches that rely upon specific active ingredients may enable better treatment personalization and greater treatment efficacy (eg, [42]). Thus, core cognitive behavioral ingredients [43] (Table 1) were delivered to patients according to their individualized needs, as was ascertained during a functional assessment. The goal of this clinician-administered, semistructured interview assessment was to identify the primary processes (eg, fear/avoidance and deficits in extinction; inactivity/sadness and deficits in response-contingent positive reinforcement) that were driving or maintaining symptoms, and then to select the therapeutic strategy that most directly targeted those processes (eg, exposure therapy and behavioral activation). Therapy was complemented by protocolized medication management as needed (modified from the STAR*D trial; [35]), delivered by psychiatry residents. Weekly CAT-MH scores made available to clinicians on a dashboard enabled continuous monitoring of treatment response and need for treatment adaptation. For example, if CAT-MH depression scores did not show improvement within 6 weeks of behavioral activation, the functional assessment was repeated, followed by possible switching to either cognitive restructuring or mindfulness-based approaches. Similarly, medication management changes were considered in light of poor response on the CAT-MH scores.

Weekly individual therapy sessions were delivered by clinical psychology doctoral students and psychiatry residents, as part of their in-house training programs. They were trained through a series of workshops and didactics on each of the evidence-based active ingredients (Table 1), consisting of didactic material, demonstrations of skills by the experts, and role-play practices of skills. They were taught to use the functional assessment and treatment decision-making algorithm to identify the principal process to target and the first-line treatment ingredient that mapped onto that target (eg, worry—cognitive restructuring; low activity/sadness—behavioral activation); and the process for monitoring symptoms to identify when a functional assessment and treatment plan should be updated. After initial training, psychology trainees participated in weekly supervision and an interdisciplinary case conference.

Psychiatry trainees were trained in the STAND medication management algorithm and received regular supervision from attending psychiatrists. Psychiatrists also participated in the same weekly interdisciplinary case conference as psychology trainees, which also included members of the campus crisis response team who could assist with coordination of outreach for high-risk participants.

**Table 1:** Matching of clinical care to patient problem area.

Problem area	Process targeted	First-line therapy module + medication as appropriate	Process targeted	Second-line therapy module + medication as appropriate
Low activity/sadness	Low response-contingent positive reinforcement	Behavioral activation (mood monitor, activity schedule, problem solve barriers, sleep schedule for barriers)	Cognitive distortions and rumination	Cognitive restructuring or mindfulness and value-driven action and problem solving
Anhedonia	Reward hyposensitivity	Pleasant event scheduling (hedonic and eudaimonic rewards), memory specificity recounting	Reward hyposensitivity	Cognitive restructuring with positive focus and cultivating positivity
Fear/phobia	Deficits in extinction, safety learning, avoidance	Exposure therapy	Negative cognitive bias and poor social skills	Cognitive restructuring, mindfulness, value-driven action or social skills training
Worry	Negative cognitive bias	Cognitive restructuring or mindfulness, value-driven action	Avoidance (experiential and in vivo)	Exposure therapy or mindfulness and value-driven action or social skills training
Sleep dysregulation	Sleep dysregulation	Brief behavioral therapy for insomnia	Negative cognitive bias	Cognitive restructuring
Trauma—fear	Deficits in extinction, safety learning, avoidance	Imaginal and in vivo exposure	Negative cognitive bias	Cognitive restructuring and impact statement
Trauma—guilt, shame, cognitive distortions	Negative cognitive bias	Trauma narrative with cognitive restructuring and impact statement	N/A^a^	N/A
Chronic suicidality, self-harm, affective instability	Low tolerance of distress	Distress tolerance skills in DBT^b^	Poor emotion regulation and interpersonal difficulties	Emotion regulation skills and interpersonal effectiveness in DBT
Mania	Circadian dysregulation	Brief behavioral therapy for insomnia	N/A	N/A
Major life stressors (any symptom profile)	Poor coping	Problem solving for controllable stressor, mindfulness, value-driven action for uncontrollable stressor	N/A	N/A
Interpersonal relations (any symptom profile)	Social skills deficits	Interpersonal effectiveness training in DBT	N/A	N/A

^a^N/A: not applicable (ie, no second-line therapy module for trauma, mania).

^b^DBT: dialectical behavioral therapy.

#### Tier Adaptation

Across all tiers, treatment was adapted according to weekly or biweekly CAT-MH scores. If participants’ scores indicated a need for a higher tier of care at any time (ie, showed a 30% worsening of symptom severity from baseline over 2 consecutive assessments), they were contacted to initiate switching to the appropriate level of care. Similarly, participants in tier 3 were switched to a lower tier when their symptoms remained consistently low (ie, in the moderate to normal range of severity following ~12-16 weeks of treatment). Thus, when participants completed treatment in tier 3 they were offered access to ThisWayUp materials from tiers 1 and 2. Moreover, within tier 3, clinicians used weekly CAT-MH scores to switch treatment strategies (such as switching from behavioral activation to cognitive restructuring).

#### Suicide Risk Monitoring

The CAT-MH [34] digitally identified participants at risk of suicide; scores above a preset threshold triggered an alert to initiate a risk management protocol. The CAT-MH includes (but is not limited to) the screener module from the Columbia Suicide Severity Rating Scale (CSSRS; [44]). The CSSRS yields a positive case as indicated by the presence of current suicide ideation *plus* current intent *or* current method *or* recent suicidal behavior (eg, suicide attempt, steps taken toward an attempt). A positive case automatically triggered a 24/7 crisis service that made up to 3 attempts to contact the participant to conduct a risk assessment and appropriate care management. All participants in the STAND program consented to be contacted by this crisis service at study entrance. Relevant STAND staff and clinicians were also informed, and could make additional outreach efforts if the crisis service outreach attempts were unsuccessful. In the event that the participant was unable to be reached by the crisis service or STAND staff and clinicians, the deployment of other community-based crisis units was considered on a case-by-case basis.

### Measures

#### Clinical Outcomes

The primary outcome measure was the CAT-MH [34]. The CAT-MH is a commercially available (Adaptive Technologies) computerized adaptive test for assessing a variety of symptoms including those of anxiety (CAT-ANX; [45]), depression (CAT-DI; [46]), and suicide risk severity (Computerized Adaptive Test-Suicide Scale [CAT-SS]; [47]). The CAT-MH scores included symptom severity as a cumulative score of endorsed symptoms (0-100) or severity categories per symptom cluster for the CAT-DI and CAT-ANX (normal, mild, moderate, and severe) based on empirically derived cut points.

Depression scores (CAT-DI severity) of <50.0 were considered normal, 50.0-65.0 mild, >65.0-75.0 moderate, and ≥75.0 severe for the majority of the recruitment period (through June 2019) [46,48], and anxiety scores (CAT-ANX severity) of <35.0 were considered normal, 35.0-50.0 mild, >50.0-65.0 moderate, and >65.0 severe [45,48]. From June 2019 to June 2020 (ie, end of recruitment), CAT-DI categories were slightly changed based on findings from [48] that led to recommendations from the CAT-MH developer team (R. D. Gibbons, PhD, personal communication, June 9, 2019). During this latter period, the threshold for mild depressive symptoms was lowered such that scores <35.0 were considered normal, and the mild range was 35.0-65.0. The CAT-MH was administered weekly (tier 3) or biweekly (tiers 0-2) across the entire course of participation in STAND. The CAT-MH categories were used to triage participants into initial tier, and were used to inform adaptation to tiers (ie, tier switching) during the next 40 weeks. Therefore, the primary outcomes gathered from the CAT-MH (ie, CAT-ANX severity score, CAT-DI severity score, and CAT-SS severity score) were the dependent variables. See [34,45-47] for details regarding the excellent psychometric properties of the CAT-ANX, CAT-DI, and CAT-SS.

#### Participant Acceptability

Participants were asked to provide feedback on their experience in the STAND program when they reached the final 40-week assessment (this assessment was initiated halfway through the study). Participants were asked to rate how logical the program seemed (on a scale from 1 to 9, where 1=not at all logical, 5=somewhat logical, and 9=very logical), to what extent the program met their expectations (on a scale from 1 to 5, where 1=completely failed to meet expectations, 3=met expectations, and 5=greatly exceeded expectations), how satisfied they were with the program (on a scale from 1 to 7, where 1=extremely dissatisfied, 4=neither satisfied nor dissatisfied, and 7=extremely satisfied), and how likely they would be to recommend the program to a friend (on a scale from 1 to 7, where 1=extremely unlikely, 4=neither likely nor unlikely, and 7=extremely likely).

### Statistical Analysis

Descriptive statistics were used to characterize the data and to identify indices of feasibility and acceptability. With regard to the primary outcome data, a series of hierarchical linear models (1 per tier per outcome measure) were conducted with time as the level 1 predictor and the outcome of interest (CAT-ANX, CAT-DI, and CAT-SS scores for tier 3, and CAT-ANX and CAT-DI scores for tiers 1 and 2). In line with an effectiveness paradigm, we included all participants who were assigned to a tier and had at least one CAT-MH datapoint, regardless of how many sessions/lessons were completed or how much missing data on the CAT-MH was observed. Parallel sets of analyses were run with and without a covariate (number of sessions/lessons completed on level 2). The survey data were not consistently collected for participants in tiers 1 and 2 after completion of the *ThisWayUp* program. Therefore, results for tiers 1 and 2 reflect CAT-MH scores over the course of *ThisWayUp* program completion only (ie, 8 weeks). Further, of the 11 participants who completed a baseline assessment for tier 0, only 5 participants completed more than 3 additional assessments. For this reason, CAT-MH scores are not reported for tier 0.

As a secondary analysis, the percentages of those within each tier who achieved at least 30% improvement on each CAT-MH index (ie, CAT-DI, CAT-ANX, and CAT-SS) were calculated among participants considered at least moderately engaged (ie, completed at least four sessions/lessons).

### Ethics Approval

The project was approved by the UCLA Institutional Review Board (approval numbers 17-001938, 16-001395, and 17-001365).

## Results

### Indices of Feasibility

#### Coach and Clinician Training

A total of 530 student coaches (including undergraduates in the resilience peer network for tier 1 and doctoral students for tier 2) were trained (to an adequate or better level, thus allowing them to deliver coaching; see [39]), and 47 psychology and psychiatry clinicians were trained to deliver psychological and psychiatric care in tier 3. For further details on student coach training and competency assessment, see [39].

#### STAND Participant Uptake, Engagement, and Adaptations

A total of 4845 unique students completed screening using the CAT-MH; 3580 out of the 4845 students who completed screening (73.89%) were eligible and offered care in tiers 1, 2, or 3; 516 out of the 3580 eligible students who were offered care (14.41%) initiated therapy in one of the tiers. As many as 327 individual suicide risk alerts were detected at screening, and additional risk assessments (after screening) were conducted for 1054 alerts indicating a risk of suicide or severely worsening depression over the course of the study.

A total of 180 students received care (ie, completed at least one lesson) in tier 1 and 197 in tier 2. Those in tier 1 completed on average 4.22 (SD 1.91) out of 6 digital therapy lessons, which was similar or better than adherence reported in previous studies [37,38]. Tier 1 participants completed on average 0.88 (SD 2.07) coaching sessions. The correlation between number of coaching sessions and digital therapy lessons completed in tier 1 was small but significant and positive (*r*=0.25, *P*<.001). Students in tier 2 completed on average 4.09 (SD 2.06) out of 6 digital therapy lessons and 2.09 (SD 2.56) group or 1-on-1 coaching sessions. The correlation between number of coaching sessions and digital therapy lessons completed in tier 2 was moderately large and significant (*r*=.54, *P*<.001). With regard to “tier switching”/adaptation, only 5 participants who were in tiers 1 or 2 were moved up to tier 3 due to a symptom worsening or suicide risk alert. A total of 139 students received clinical care in tier 3 (ie, attended at least one therapy session), and attended on average 13.86 (SD 7.94) therapy sessions. Following the acute course of tier 3 treatment, all tier 3 participants were offered online CBT via *ThisWayUp* (tier 1 without coaching).

Demographic, diagnostic, and baseline clinical data for tiers 0-3 are presented in Table 2.

**Table 2:** Demographic characteristics.

Characteristics^a^		Tier 0	Tier 1	Tier 2	Tier 3
Completed baseline assessment, n		11	225	239	144
Age (years), mean (SD)		22.91 (6.56)	23.01 (5.81)	21.93 (4.47)	21.14 (3.36)
**Assigned sex at birth, n**					
	Female	5	160	184	107
	Male	6	63	53	36
**Current gender identity, n**					
	Female	5	159	181	105
	Male	6	61	51	35
	Transgender	0	1	2	0
	Does not identify as either	0	2	3	3
**Sexual orientation^b^, n**					
	Asexual	0	1	0	1
	Bisexual	0	12	25	27
	Heterosexual/straight	8	49	82	81
	Homosexual, gay or lesbian	1	2	5	7
	Queer	0	3	6	6
	Questioning or unsure	1	8	12	9
	Not listed	1	0	3	0
	Prefers not to answer	0	1	2	5
**Marital status, n**					
	Single, never married	10	195	211	131
	Living with partner	1	10	9	4
	Domestic partnership	0	0	2	1
	Married	0	14	11	4
	Separated	0	0	0	0
	Divorced	0	2	0	1
	Do not know	0	0	1	1
	Prefers not to answer	0	2	2	1
**First language, n**					
	English	11	159	161	104
	Spanish	0	22	23	15
	Other	0	42	53	24
**Country of birth, n**					
	United States	10	171	174	111
	Other	1	52	63	32
**Immigration status, n**					
	Domestic	11	194	201	126
	International	0	25	31	13
	Undocumented	0	4	4	2
	Other	0	0	0	0
	Prefers not to answer	0	0	1	2
**Racioethnic group, n**					
	Hispanic	1	40	59	36
	Non-Hispanic White	3	85	71	35
	Non-Hispanic Black	2	4	10	4
	Non-Hispanic Asian	4	72	71	52
	Non-Hispanic multiple	1	17	17	12
	Non-Hispanic Native American/Pacific Islander	0	0	0	0
	Unknown	0	5	9	4
**Highest level of education, n**					
	High school graduate	2	34	44	43
	General educational development or equivalent	0	1	0	0
	Some college	4	85	96	59
	Associate’s degree (occupational, technical, or vocational)	0	1	3	0
	Associate’s degree (academic program)	0	12	27	14
	Bachelor’s degree	5	66	46	18
	Master’s degree	0	23	18	8
	Professional degree	0	1	2	0
	PhD	0	0	1	0
	Prefers not to answer	0	0	0	1
**Employment, n**					
	Working	3	20	26	16
	Only temporarily laid off, sick leave, or maternity leave	0	0	0	1
	Looking for work, unemployed	0	6	6	2
	Disabled, permanently or temporarily	0	0	0	1
	Student	8	194	201	121
	Other	0	3	4	2
**Student enrollment status, n**					
	Full time	10	215	229	138
	Part-time	0	4	7	5
**College level, n**					
	Freshman	3	30	35	34
	Sophomore	0	29	41	25
	Junior	1	42	61	32
	Senior	2	37	36	26
	Graduate student	4	70	55	22
	Professional student	0	9	5	4
	Other	0	2	3	0
**Baseline CAT-MH^c^ Depression**					
	Depression severity, mean (SD)	33.63 (9.04)	49.90 (11.44)	59.96 (12.25)	73.21 (11.32)
	Minimal to none depression, n	10	96	36	5
	Mild depression, n	1	109	116	21
	Moderate depression, n	N/A^d^	19	65	61
	Severe depression, n	N/A	N/A	21	57
**Baseline CAT-MH Anxiety**					
	Anxiety severity, mean (SD)	18.42 (12.74)	39.65 (12.51)	55.60 (14.37)	60.14 (16.10)
	Minimal to none anxiety, n	10	82	19	8
	Mild anxiety, n	1	104	74	32
	Moderate anxiety, n	N/A	33	79	45
	Severe anxiety, n	N/A	6	66	59
**Baseline CAT-MH Suicidality**					
	Suicidal ideation diagnosis, n	N/A	2	6	24
	Suicidality severity, mean (SD)	18.49 (12.95)	39.62 (12.67)	50.29 (11.99)	62.19 (11.76)

^a^Participants were able to enroll in the study at both wave 1 and wave 2 of recruitment. Therefore, some participants may be represented twice if they chose to reenroll in the study.

^b^This question was introduced partway through the study, therefore not all participants had the opportunity to respond.

^c^CAT-MH: Computer Adaptive Test for Mental Health.

^d^N/A: not applicable.

### STAND Clinical Outcomes (Preliminary Effectiveness)

#### Overview

**Figure 3:**
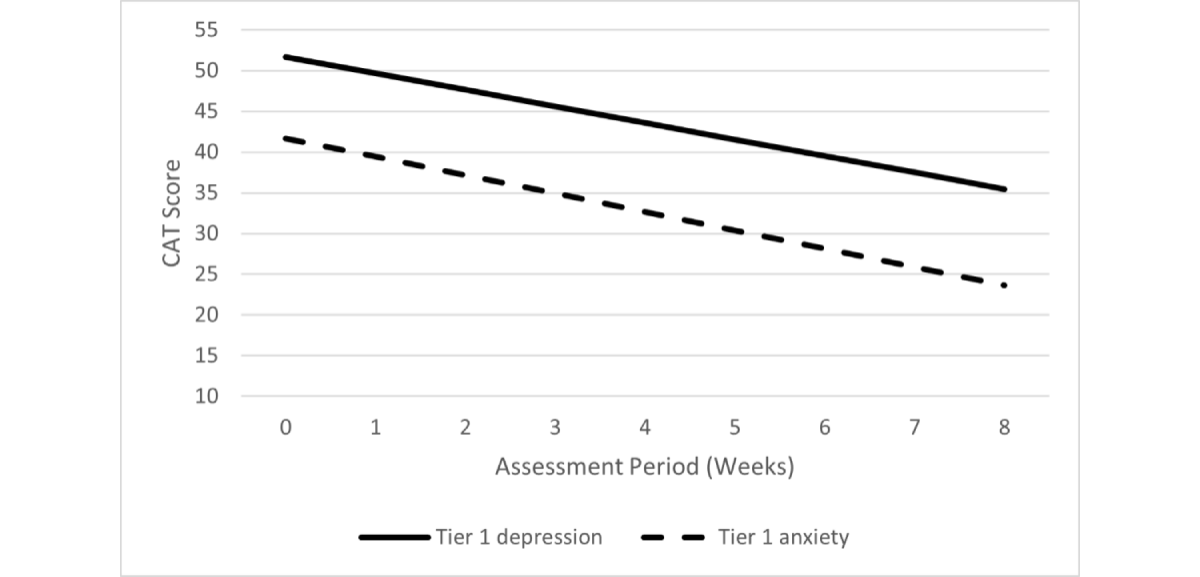
Tier 1 changes in CAT-DI and CAT-ANX scores over time. CAT-ANX: Computerized Adaptive Test-Anxiety Inventory; CAT-DI: Computerized Adaptive Test-Depression Inventory; SI: suicidal ideation.

**Figure 4:**
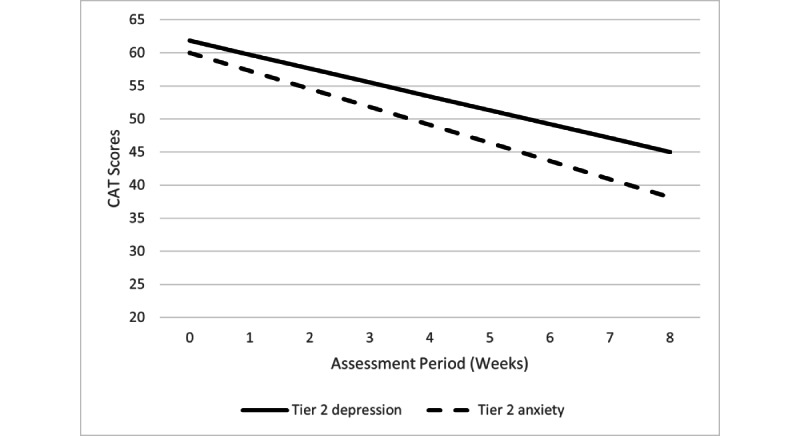
Tier 2 changes in CAT-DI and CAT-ANX scores over time. CAT-ANX: Computerized Adaptive Test-Anxiety Inventory; CAT-DI: Computerized Adaptive Test-Depression Inventory.

**Figure 5:**
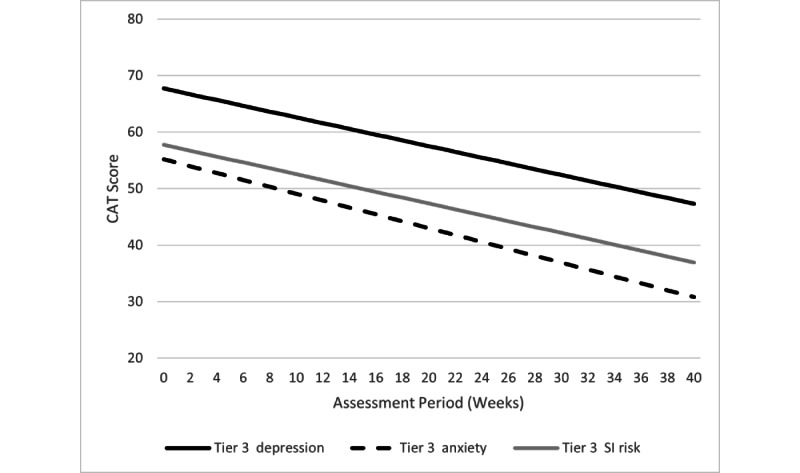
Tier 3 changes in CAT-DI, CAT-ANX, and CAT-SS scores over time. CAT-ANX: Computerized Adaptive Test-Anxiety Inventory; CAT-DI: Computerized Adaptive Test-Depression Inventory; CAT-SS: Computerized Adaptive Test-Suicide Scale.

There was no difference in the pattern or significance of any findings when the covariate (ie, number of sessions/lessons completed) was included. Therefore, analyses without statistically adjusting for the number of sessions/lessons completed are reported below as a conservative approach. Specific trajectories for each tier/outcome combination are described below in more detail. Figures 3 and 4 show the decline slopes for the CAT-ANX and CAT-DI within each tier, and Figure 5 shows the CAT-SS decline slope in tier 3.

#### Tier 1

The CAT-DI and CAT-ANX scores significantly declined over the course of STAND participation (*b*=–2.03, t_732_=–10.71, *P*<.001 and *b=*–2.26, t_732_=–12.47, *P*<.001, respectively). Based on the regression lines, scores moved from the mild to normal range for both depression and anxiety severity (Figure 3). Among participants who completed at least four digital therapy lessons (n=110), 51 participants (46.4%) showed ≥30% improvement in CAT-DI scores and 62 participants (56.4%) showed ≥30% improvement in CAT-ANX scores, notable for a sample likely to have a floor effect, with minimal room for significant symptom improvement.

#### Tier 2

The CAT-DI and CAT-ANX scores significantly declined over the course of STAND participation (*b*=–2.10, t_696_=–9.64, *P*<.001 and *b=*–2.73, t_696_=–12.42, *P*<.001, respectively). As shown in Figure 4, although scores for depression began and ended in the mild range (following the adjustment of categories in June 2019), they moved from the top of the mild range into the lower end of the mild range (and into what was considered the normal range prior to June 2019). Anxiety severity scores moved from the moderate range to the mild range. Among participants who completed at least four digital therapy lessons (n=106), 33 participants (31.1%) showed ≥30% improvement in CAT-DI scores and 47 participants (44.3%) showed ≥30% improvement in CAT-ANX scores.

#### Tier 3

The CAT-DI and CAT-ANX scores significantly declined over the course of STAND participation (*b*=–0.61, t_2805_=–11.91, *P*<.001 and *b=*–0.51, t_2805_=–9.64, *P*<.001, respectively). The CAT-SS severity also declined over time (*b=*–0.52, t_2805_=–11.05, *P*<.001). As depicted in Figure 5, the regression line shows depression scores moved from the moderate range to the mild range (and what was considered the normal range prior to June 2019) and anxiety scores moved from the moderate range to the normal range. Among participants who completed at least four therapy sessions (n=131), 74 participants (56.5%) showed ≥30% improvement in CAT-DI scores, 81 participants (61.8%) showed ≥30% improvement in CAT-ANX scores, and 78 participants (59.5%) showed ≥30% improvement in CAT-SS scores.

### Participant Acceptability

Results on the subsample (n=79) who completed the satisfaction survey are reported in Table 3. In sum, participants across tiers 1-3 rated their care as highly logical (scores ranging from 6.65 to 7.60 out of 9), that the program succeeded in meeting their expectations (scores ranging from 3.47 to 3.95 out of 5), that they were satisfied with the program (scores ranging from 5.24 to 5.90 out of 7), and that they would recommend it to a friend (scores ranging from 5.47 to 6.14 out of 7). Tier 0 participants also rated these categories highly but scores should be interpreted with caution, given the small sample size in tier 0 (Table 3).

**Table 3:** Program feedback.

Variables	Tier 0	Tier 1	Tier 2	Tier 3
Participants, n	3	17	17	42
Logical, mean (SD)	7.33 (0.58)	6.65 (2.23)	6.59 (1.28)	7.60 (1.62)
Expectation, mean (SD)	3.00 (0.00)	3.47 (1.01)	3.76 (0.83)	3.95 (1.04)
Satisfaction, mean (SD)	4.67 (0.58)	5.24 (1.56)	5.47 (1.01)	5.90 (1.25)
Recommendation, mean (SD)	4.67 (0.58)	5.65 (1.37)	5.47 (0.94)	6.14 (1.18)

## Discussion

### Summary of Outcomes

This preliminary study demonstrated that STAND, a technology-assisted and scalable system of care, is feasible and effective on a college campus. STAND facilitated the deployment of a significant number of suicide risk assessments as needed in real-time, a critical index of risk prevention. As part of the STAND model, a large number of coaches (n=530) and clinicians (n=47; in psychiatry and psychology) were trained to provide support for or deliver evidence-based treatment for anxiety and depression, respectively. Care was delivered to 516 UCLA students (ie, those who completed at least one session of therapy/digital therapy lesson), who, on average, experienced significant reductions in their anxiety and depression symptoms across all tiers. Participant acceptability on the satisfaction survey revealed moderately high ratings of satisfaction with participants’ assigned tier. Importantly, the stratified model of initial triaging to the appropriate level of care was successful in that the vast majority of participants showed significant improvement in their originally assigned tier. Indeed, only 5 participants required a higher level of care during their initial participation in a lower tier.

Although only a small percentage of participants who completed the initial CAT-MH screening assessment and were offered care actually initiated care in one of the tiers (516/3580, 14.41%), there is a wide range of uptake reported in similar digital interventions, with the rate in this study falling within that range [5,49,50], and promising outcomes emerged among those who did participate. Additionally, a prior analysis of racial/ethnic differences in STAND treatment uptake midway through the recruitment period found that students from ethnic/racial minority groups were not less likely to enroll or engage in treatment compared with non-Hispanic White students (see [51]), indicating that STAND has the potential to reach and be acceptable to a diverse student population, which is important for our current work that aims to implement STAND in underserved communities. Of note, although significant improvement over time was observed across all outcome measures within each tier, a relatively lower percentage of participants in tier 2 showed clinically meaningful change across outcomes than tiers 1 and 3, suggesting that there may be some participants who were assigned to tier 2 who may have benefited more from a higher level of care. Our current work is examining whether including other variables (eg, life stress, trauma, demographics) in multivariate predictive models alongside symptom severity can improve upon and personalize tier triaging to improve clinical outcomes.

Taken together, these findings support the feasibility, acceptability, and preliminary effectiveness of STAND; and this initial demonstration of the STAND model of care provided an opportunity to identify areas for improvement and refinement of the model, including increasing engagement and retention in care.

### Limitations

The data presented represent the initial implementation of this system in an open trial. Therefore, conclusions about the effectiveness of STAND compared with another treatment cannot be drawn. Further, as is common practice with initial implementations that aim to iteratively refine and improve upon methodology, some minor data-driven changes were made throughout the course of the study (eg, changing CAT-DI categories based on incoming psychometric information, shifting from group to individual coaching sessions). Another limitation was the low response rate, particularly in tier 0 and following the *ThisWayUp* intervention in tiers 1 and 2. This low response rate precludes our ability to evaluate the longer-term outcome in the lower tiers. Finally, with regard to booster treatment during the monitoring period following the initial acute phase of care, in the present demonstration of STAND, we opted to provide the digital therapy to all participants who had completed tier 3 treatment. However, we did not track usage during this preventative/maintenance phase. Future work we have planned will more precisely identify those who may need this additional booster and at what point in their trajectory. Finally, we did not systematically collect demographic data (eg, gender, race/ethnicity) on coaches and clinicians, which would have been interesting to examine both descriptively and correlations between participant/clinician match on demographic factors and clinical outcomes/engagement. Our current work is now collecting these data on coaches in tier 2 as well as conducting a randomized trial to examine the potential impact of racial/ethnic matching between coaches and participants on engagement and clinical outcomes.

### Lessons Learned and Current Directions in STAND

Two notable observations in this initial implementation of STAND were the low initial uptake of STAND services, and the relatively modest ongoing engagement in the digital therapy tiers. With regard to the former, the vast minority of students who were screened and eligible to receive services in STAND actually engaged in care. Despite our multipronged approach to recruitment, and the possibility that a lack of financial incentive to participants in the study may have contributed to some extent in low uptake, improvements to our recruitment strategies are clearly needed. Specifically, additional research is needed to identify recruitment strategies (possibly tailored to each new population that adopts STAND) that provide messaging that resonates with the student population. We are currently partnering with social marketing experts to use inclusive, nonstigmatizing messaging and improve upon our outreach efforts; and current research projects underway are seeking feedback from students who did not engage in care to identify barriers and develop solutions.

With regard to the latter, consistent with prior evidence [49,52], retention in digital therapy was somewhat problematic, and utilization of coaching support was particularly low. This is a ubiquitous challenge in digital therapy research for which creative solutions are being developed and evaluated (eg, the use of gamification, avatars, personalization, feedback, and individual support; see [53] for a review). Although our digital therapy approach already incorporates several of these features, we have made several changes to improve retention in digital therapy and coaching, which are currently being implemented and evaluated in ongoing trials. These include automatic scheduling of all digital therapy participants for coaching sessions, rather than making it an add-on option, and personalizing digital therapy by switching from a unified, one-size-fits-all approach to a modularized suite of digital components that are selected using embedded measurement systems to match an individual’s presenting concerns. The change to a personalized approach to module selection is expected to increase retention given evidence for students to prefer tailored online therapy [54]. Current studies are also underway to develop and evaluate engagement strategies (with an emphasis on culturally responsive messaging) and to implement text messaging as a method to increase engagement.

Other changes to our current iteration of STAND include collapsing tiers 1 and 2 into 1 tier (tier 2), with streamlined coach training and supervision, as well as provision of a self-guided wellness digital program in the original tier 0 (now called tier 1). Given that we found such low engagement in tier 0 (the monitoring-only program), we were unable to analyze data collected from tier 0 participants. Possibly, students did not see the benefit of participating in a monitoring-only program when they had minimal to no symptoms (and were not being financially compensated). Our expectation is that by providing digital prevention tools framed as stress management and wellness, students with no symptoms will be more interested in participating in STAND.

### Conclusions

The need for effective and scalable treatments for depression and anxiety is enormous given the high (and rising) rates of these disorders and pervasive lack of availability of accessible, evidence-based care. The Screening and Treatment of Anxiety and Depression (STAND) system of care was developed to address this need. STAND is based on the principles of stratified stepped care, adaptation of care in response to continuous symptom monitoring, and routine detection and prevention of suicidality. The STAND system of care was tested in a large sample of UCLA students, where it led to significant reductions in depression and anxiety symptoms and suicide risk. Efforts are underway to continue to refine and enhance the STAND system of care, while increasing its reach, with an emphasis on reaching underserved, diverse populations, conducting randomized clinical trials to identify the optimal ways to deliver STAND, and developing a pathway for sustainability of STAND as it is rolled out on a larger scale. These sustainability efforts will include stakeholder input and economic analyses to identify the specific resources needed to carry out each component of STAND, including ensuring that a sufficient clinical and coaching workforce is available and trained to fidelity in partnership with a college campus that wishes to implement STAND.

## Abbreviations

CAT-ANX: Computerized Adaptive Test-Anxiety Inventory
CAT-DI: Computerized Adaptive Test-Depression Inventory
CAT-MH: Computer Adaptive Test for Mental Health
CAT-SS: Computerized Adaptive Test-Suicide Scale
CSSRS: Columbia Suicide Severity Rating Scale
STAND: Screening and Treatment for Anxiety and Depression
UCLA: University of California, Los Angeles
